# Machine learning and dyslexia: Classification of individual structural neuro-imaging scans of students with and without dyslexia

**DOI:** 10.1016/j.nicl.2016.03.014

**Published:** 2016-03-29

**Authors:** P. Tamboer, H.C.M. Vorst, S. Ghebreab, H.S. Scholte

**Affiliations:** University of Amsterdam, Faculty of Social and Behavioural Sciences, Weesperplein 4, 1018XA Amsterdam, The Netherlands

**Keywords:** SVM, support vector machine, VBM, voxel-based morphometry, GM, gray matter, VWFA, visual word form area, LIPL, left inferior parietal lobule, LOFG, left occipital fusiform gyrus, ROFG, right occipital fusiform gyrus, Dyslexia, MRI, SVM classification, Gray matter, VWFA

## Abstract

Meta-analytic studies suggest that dyslexia is characterized by subtle and spatially distributed variations in brain anatomy, although many variations failed to be significant after corrections of multiple comparisons. To circumvent issues of significance which are characteristic for conventional analysis techniques, and to provide predictive value, we applied a machine learning technique – support vector machine – to differentiate between subjects with and without dyslexia.

In a sample of 22 students with dyslexia (20 women) and 27 students without dyslexia (25 women) (18–21 years), a classification performance of 80% (*p* < 0.001; d-prime = 1.67) was achieved on the basis of differences in gray matter (sensitivity 82%, specificity 78%). The voxels that were most reliable for classification were found in the left occipital fusiform gyrus (LOFG), in the right occipital fusiform gyrus (ROFG), and in the left inferior parietal lobule (LIPL). Additionally, we found that classification certainty (e.g. the percentage of times a subject was correctly classified) correlated with severity of dyslexia (r = 0.47). Furthermore, various significant correlations were found between the three anatomical regions and behavioural measures of spelling, phonology and whole-word-reading. No correlations were found with behavioural measures of short-term memory and visual/attentional confusion. These data indicate that the LOFG, ROFG and the LIPL are neuro-endophenotype and potentially biomarkers for types of dyslexia related to reading, spelling and phonology.

In a second and independent sample of 876 young adults of a general population, the trained classifier of the first sample was tested, resulting in a classification performance of 59% (*p* = 0.07; d-prime = 0.65). This decline in classification performance resulted from a large percentage of false alarms. This study provided support for the use of machine learning in anatomical brain imaging.

## Highlights

• Abnormalities in brain anatomy of dyslexics were investigated with machine learning.
• Using support vector machine, dyslexic and non-dyslexic students were classified.
• A classification performance of 80% was achieved, based on differences in gray matter.
• A classification performance of 59% was achieved in an independent sample.
• Relevant areas were the left and right OFG and the left IPL.

## 1 Introduction

Dyslexia is usually defined as a specific reading disorder characterized by a specific and significant impairment in the development of reading skills that are unrelated to problems with visual acuity, schooling or overall mental development (World Health Organisation, 2010). For (sub-) groups of dyslexics, reading difficulties have been related to various symptoms of which the most frequently reported are related to phonological difficulties although, in recent years, visual/attentional deficits are reported frequently as well (e.g. Ramus and Ahissar, 2012). Generally, it is assumed that early learning delays cannot be overcome completely despite remedial teaching programs, and that these learning delays interfere with academic achievement into adulthood for most of the dyslexics which are estimated to represent 5% to 15% of the population.

Reliable diagnoses can currently only be determined behaviourally and after some years of education, when the discrepancy between normal cognitive and reading abilities becomes visible. Alternatively, researchers have been searching for biomarkers of dyslexia using MRI or fMRI. Meta-analyses showed that these differences do exist (Richlan et al., 2011, Richlan et al., 2012, Vandermosten et al., 2012), although many findings failed to be significant after corrections of multiple comparisons.

A potentially more powerful technique than the univariate voxel-wise evaluation and correction of multiple comparisons are multivariate classification techniques from machine learning. This technique has recently successfully been applied in several clinical neuroimaging studies. For instance, a high accuracy rate of 90% has been reported for discriminating major depressive disorder and controls (Mwangi et al., 2012). An accuracy rate of 81% has been found for autism (Ecker et al., 2010).

Also with regard to dyslexia, this classification approach has been applied. In a study of Hoeft et al. (2011), a multivariate pattern analysis of brain activation during a reading task over the whole brain using linear support vector machine and cross-validation, showed that reading gains over a 2.5 year period in children with dyslexia can be predicted with > 90% classification accuracy. A study of Tanaka et al. (2011) showed that in two samples of typical and poor reading children 79% and 80% were classified correctly using leave-one-out linear SVM analyses of brain activation during phonological processing. In a study of Pernet et al. (2009), classification of dyslexic readers brains resulted in dyslexics falling outside the 95% confidence boundaries of the controls in two areas (the right cerebellar declive and the right lentiform nucleus).

The aim of this study is to investigate whether young adults with and without dyslexia can reliably be classified based on anatomical differences. We examined neuro-anatomical networks involved in dyslexia using a whole-brain classification employing SVM and cross-validation. We used the T_1_-weighted magnetic resonance images of GM structure of a sample of 22 students with dyslexia and 27 students without dyslexia for acquiring a trained classifier. Next, we determined which voxels were involved with the correct classification. Furthermore, we explored to what degree these results can be used to investigate the relation between different cognitive aspects of dyslexia and neural substrates. We also tested the reliability of the trained classifier in an independent sample of 876 young adults.

## 2 Materials and methods

### 2.1 Subjects & procedure

The first sample – used to find a trained classifier – consisted of 22 students with dyslexia (20 women; 4 left-handed; mean age 20.7 years, SD 1.8 years) and 27 students without dyslexia (25 women; 4 left-handed; mean age 20.3 years, SD 0.9 years). All participating subjects were first-year psychology students, native Dutch speakers, had at least twelve years of school education, were free from medical or psychiatric diseases and had no history of sensory deficits or head trauma. None of the participants had a diagnosis of ADHD. Handedness was assessed with a short self-report questionnaire, which included questions about writing hand, general hand preference, as well as 20 specific questions. There were no students with inconsistent reports which could indicate being ambidextrous.

The 49 students of the first sample were invited to participate in the present study by mail and telephone. The students gave informed written consent and were debriefed afterwards. All participants had the option to choose between acquiring participation points required for the first year of study, or a financial reward. This study was approved by the ethics committee at the University of Amsterdam.

The second sample – used to test the trained classifier – consisted of young adults of a general population. Brain data of this sample were available for various studies at the University of Amsterdam. We excluded participants with a serious medical condition, with a diagnosis of autism spectrum disorder and participants using psychiatric drugs or psychiatric medicine. The remaining sample consisted of 876 subjects who were native Dutch speakers and who had at least twelve years of school education. Of this sample, 60 (7%) subjects (27 women; mean age 22.5 years, SD 1.6 years) were diagnosed with dyslexia whilst attending school, and 816 subjects (433 women; mean age 22.9 years, SD 1.7 years) had no reported history of dyslexia.

### 2.2 Neuropsychological Assessment

The first sample was acquired from a sample of 480 students who participated in a previous study (Tamboer et al., 2014a). In that study, dyslexia and non-dyslexia was assessed using three sources of information: (1) a history of language difficulties, (2) a self-report of language difficulties, and (3) a test-battery measuring numerous abilities such as spelling, reading, pseudoword reading, phonology, attention, and short-term memory. Severity of dyslexia was determined with a regression formula which consisted of 13 test items and 10 self-report questions, and which classified all subjects with and without dyslexia correctly. In a follow-up study (Tamboer et al., 2014b), five behavioural factors accompanying dyslexia were determined using exploratory and confirmatory factor analyses. On the basis of these analyses we acquired five Z-transformed sum scores: spelling, phonology, short-term memory, visual/attentional confusion, and whole-word reading.

We assumed that intelligence of all participants was within the normal range because all had finished the highest level of secondary school education in the Netherlands. Group differences of intelligence were analysed as follows. In the original sample of 480 students, we performed factor analyses over six subtests of a cognitive battery that was based on the Structure of Intellect Model of Guilford and Raven Progressive Matrices for a better interpretation of various aspects of intelligence. Three factors (non-verbal intelligence, speed of numeric processing, vocabulary) were extracted and factor scores were acquired with a mean of zero and standard deviation of one. The smaller sample of the present study shows small deviations from the mean and SD of 1 because this was a selection of the original sample. In the present sample, the groups did not differ on the three aspects of general intelligence. Furthermore, no differences were found on school grades of English language, mathematics, and other courses. However, the dyslexic group had compared to the non-dyslexic group lower final school grades of Dutch language and other languages such as French or German. We conclude that the groups did not differ in terms of general intelligence. Specific details can be found under *Supplementary Information*.

The data of the second sample were collected to be used in various studies regarding brain correlates accompanying various developmental disorders. The subjects of this sample were not tested for dyslexia, because the present study was performed after the collection of data. Available was a large self-report questionnaire which included two questions about dyslexia. One question was whether the subjects had an official certificate of dyslexia and a second question was whether a subject was tested for dyslexia whilst attending school.

### 2.3 Image acquisition and preprocessing

For both samples, we used the standard population acquisition protocol of the Spinoza Centre for NeuroImaging in Amsterdam. We acquired three 3DT1 whole-brain scans for each subject (3D T1, Turbo Field Echo sequences, voxel size = 1 mm^3^, FOV = 256^2 mm, 160 slices, FA = 8**°**, TE = 3.81 ms, TR = 8.24 ms), using a 3 T Philips Achieva scanner with a 32 channel headcoil. Each sequence lasted approximately 6 min to acquire. The three T1 scans were aligned to the 2nd recorded T1 scan and subsequently averaged. Each averaged brain was manually inspected and subsequently placed in a common space using VBM (Good et al., 2001) as implemented in FSL (Smith et al., 2004).

First, structural images were brain-extracted. Next, tissue-type segmentation was carried out using FAST4 (Zhang et al., 2001). The resulting GM partial volume images were then aligned to MNI152 standard space using the affine registration. The resulting images were averaged to create a study-specific template, to which the original GM images were then non-linearly re-registered with a method that uses a B-spline representation of the registration warp field (Rueckert et al., 1999). The registered partial volume images were then modulated (to correct for local expansion or contraction) by dividing by the Jacobian of the warp field. The modulated segmented images were then smoothed with an isotropic Gaussian kernel with a kernel of 4 mm.

### 2.4 Pattern classification

**Fig. 1:**
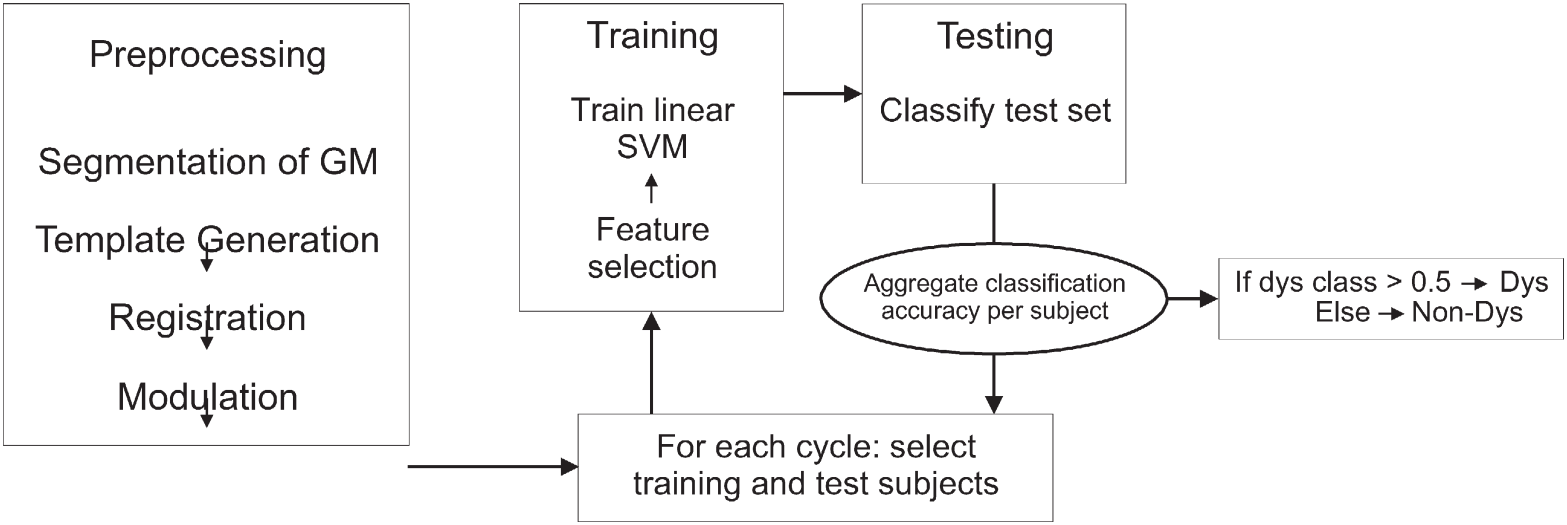
Classification scheme.

We used SVM to train a classifier to distinguish between subjects with and without dyslexia of the first sample (http://www.csie.ntu.edu.tw/~cjlin/libsvm/). The SVM classifier was trained on using 21 randomly selected subjects with dyslexia (of 22) and 21 randomly selected subjects without dyslexia (of 27). The voxels used during the training stage were determined by subtracting the average VBM transformed brain of the selected controls from the average VBM transformed brain of the dyslexia group and z-transforming the resulting difference image. Next, we estimated the linear hyperplane that maximally separates the subjects with and without dyslexia using those voxels that surpassed a z-threshold of 3, 3.5, 4 and 4.5. We used the default values for training a linear classifier, one-class classifier. This procedure yielded four classifiers which performance was evaluated using the one remaining dyslexic subject and one of the (randomly selected) six controls and we repeated this procedure 10,000 times. Thus, we applied a cross-validation procedure in which each subject was classified many times on the basis of a hyperplane acquired with 21 subjects with and 21 subjects without dyslexia. The z-threshold of 4 and 4.5 yielded the highest classification performance and we subsequently also tested a threshold value of 4.25. This z-value controls how many voxels are used for classification and resulted in these conditions into a value of 759 voxels. The procedure using the z-threshold of 4.25 yielded the highest classification performance and only these results were used for subsequent analysis. This result yielded many classifications of each subject as either dyslexic or control that could either be correct or incorrect. See Fig. 1 for the classification scheme.

### 2.5 Analyses

For each subject, group membership was determined by the majority of hits or misses. Thus, when the proportion of hits for a particular subject was ≥ 0.5, the subject was considered correctly classified. This also yielded, per subject, a continuous score representing the classification accuracy for each subject. The prediction accuracy of the trained classifier was estimated with the proportion of correctly classified subjects. We quantified the estimated sensitivity with the proportion of correctly classified subjects with dyslexia, and the estimated specificity with the proportion correctly classified subjects without dyslexia. We calculated d-prime with Z (proportion hits) – Z (proportion false alarms).

In a second analysis, we repeated the classification procedure described in the previous paragraph but using 20% of the selected voxels per iteration. For each voxel, we subsequently scored the percentage of times a classification was successful for that drawing of voxels. This yielded after 100,000 iterations, for each voxel, a count for the number of times that a voxel was selected and the number of times that this selection resulted in a correct classification. In this way we expressed the importance of a voxel for classifying dyslexia.

Next, we examined the relation between brain regions of the voxels that were most reliable for classification and the behavioural measures (spelling, phonology, short-term memory, whole-word reading and visual/attentional confusion) with bivariate Pearson correlations. We also calculated the Pearson correlation between the classification accuracy for each subject and severity of dyslexia.

In the second sample, group membership of each subject was determined by the trained classifier which was acquired with the first sample. The accuracy of the trained classifier in this sample was estimated with the proportion of correctly classified subjects. We quantified the estimated sensitivity with the proportion of correctly classified subjects with dyslexia, and the estimated specificity with the proportion correctly classified subjects without dyslexia. We calculated d-prime with Z (proportion hits) – Z (proportion false alarms).

## 3 Results

### 3.1 Classification of subjects with and without dyslexia (first sample)

The SVM technique resulted in a total prediction accuracy of 39 / 49 = 80%. Permutation testing, in which we repeated this entire procedure but now with permuted labels, revealed that in 1000 simulations this level of accuracy was never reached yielding a significance of *p* < 0.001. Furthermore, we found a sensitivity (proportion correctly classified subjects with dyslexia) of 18 / 22 = 82%, and a specificity (proportion correctly classified subjects without dyslexia) of 21 / 27 = 78%. We found that d-prime = 1.67. See Table 1. Positive predictive value (proportion of all subjects classified as dyslexic who have in fact dyslexia) was 75% and negative predictive value (proportion of all subjects classified as not dyslexic who have in fact no dyslexia) was 84%.

### 3.2 Mean classification accuracy (first sample)

For each subject separately, the accuracy of the prediction was calculated with the proportion hits or – for subjects incorrectly classified – misses. This resulted in a continuous score representing the classification accuracy for each subject, ranging from 0.51 to 1.00. In the whole sample the mean of this classification accuracy was 0.89. We also calculated the mean classification accuracy for four subgroups. The correctly classified dyslexics had a mean classification accuracy of 0.87 (SD 0.13). The false negatives had a mean classification accuracy of 0.87 (SD 0.15). The correctly classified non-dyslexics had a mean classification accuracy of 0.90 (SD 0.16). The false positives had a mean classification accuracy of 0.94 (SD 0.12). One conclusion was that the correctly predicted subjects were correctly classified in a large majority of trials. A second conclusion was that the false negatives and the false positives were correctly classified as being false negatives and false positives in a large majority of trials as well. In other words, the subjects who were incorrectly classified were consistently incorrectly classified in a large majority of trials, while more inconsistency over trials could have been expected. Simple explanations can be ruled out because we found that left-handedness, gender, and age had no influence on prediction accuracy. We also found no differences on factors of intelligence, school grades, factors of dyslexia, and severity of dyslexia between the groups of correctly and incorrectly classified subjects with dyslexia and between the groups of correctly and incorrectly classified subjects without dyslexia.

### 3.3 Anatomical classifier (first sample)

**Fig. 2:**
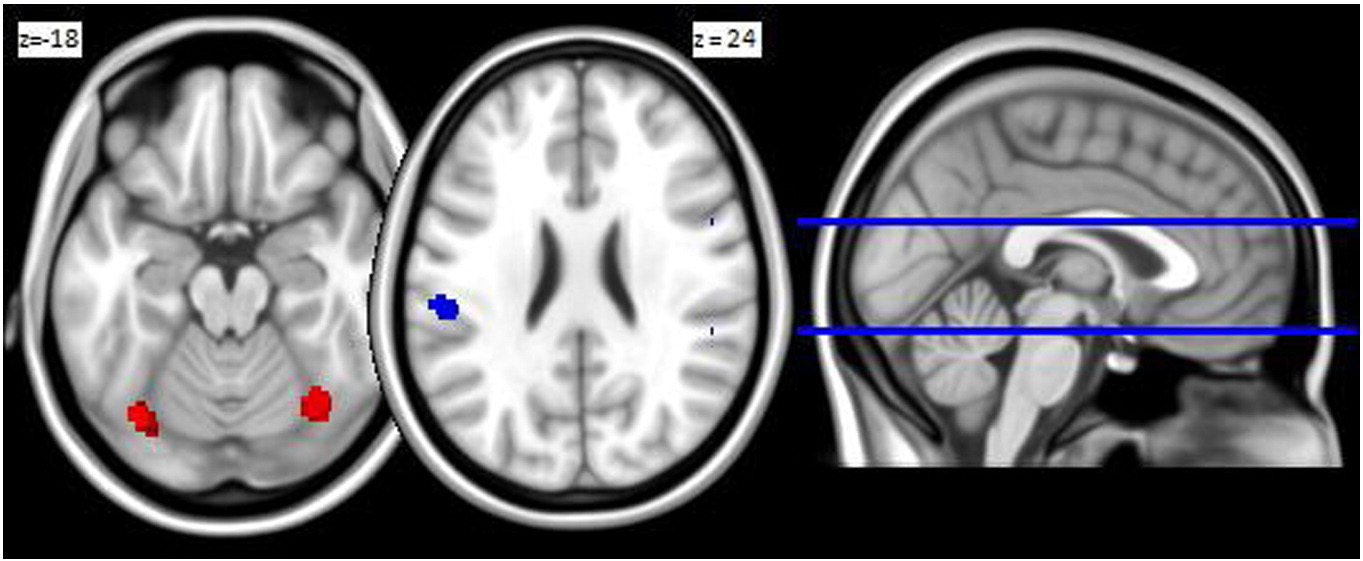
Regions with voxels involved in discriminating between subjects with and without dyslexia. Post hoc analysis revealed that the region in blue (LIPL) is smaller in subjects with dyslexia and that the regions in red (LOFG and ROFG) are larger in subjects with dyslexia. (For interpretation of the references to color in this figure legend, the reader is referred to the web version of this article.)

Fig. 2 shows three brain regions of GM (averaged between trials), which discriminated between subjects with and without dyslexia. One cluster of reduced GM volume for subjects with dyslexia was found in the LIPL (65 voxels; − 53, − 28, 24). Two clusters of augmented GM volume for subjects with dyslexia were found bilateral in the LOFG (150 voxels; − 35, − 72, − 21), and in the ROFG (187 voxels; 35, − 67, − 19). Table 2 presents the coordinates of the clusters and the direction of the differences between the groups of subjects with and without dyslexia on GM volume of the three clusters. These differences are statistically not relevant, but evaluate the relative contribution of the separate regions to the overall classification of subjects with and without dyslexia.

### 3.4 Correlation MCA – severity of dyslexia (first sample)

We linearly transformed the mean classification accuracy of each subject to a continuous score that represented severity of dyslexia according to the classifier, ranging from 0 (no dyslexia) to 1 (severe dyslexia). This score was correlated with a behavioural representation of severity of dyslexia (e.g. a regression score). We found a correlation of r = 0.47 (*p* = 0.0007).

### 3.5 Correlations between GM indices and symptoms of dyslexia (first sample)

Table 3 summarizes correlations between GM volumes of the three clusters with measures and severity of dyslexia. There are four main findings. First, severity of dyslexia correlates significantly with GM volume in the ROFG and LOFG, meaning that dyslexics have higher GM volume in those areas. Second, spelling (good performance) correlates significantly negative with GM volume in the LOFG and significantly positive with GM volume in the LIPL. Third, whole-word-reading (good performance) correlates significantly negative with GM volume in the LOFG. Fourth, phonology (good performance) correlates significantly positive with GM volume in the LIPL. No significant correlations were found for short-term memory and visual/attentional confusion. Scatter plots of the significant correlations are presented in *Supplementary Information*.

### 3.6 Classification of subjects of the second sample

The anatomical classifier of the first sample resulted in a total prediction accuracy of 59% in the second sample. Permutation testing, in which we shuffled the labels from Sample 1, revealed that the classification is lower than the observed 59% in 93% of the cases yielding a significance of *p* = 0.07. Furthermore, we found sensitivity (proportion correctly classified subjects with dyslexia) of 67%, and specificity (proportion correctly classified subjects without dyslexia) of 59%. We found that d-prime = 0.65. See Table 1. In this sample, dyslexia was overpredicted with a total number of predicted subjects with dyslexia of 43%. Positive predictive value (proportion of all subjects classified as dyslexic who have in fact dyslexia) was 11% and negative predictive value (proportion of all subjects classified as not dyslexic who have in fact no dyslexia) was 96%. We also found that total prediction accuracy did not improve when selecting the same age range as in the first sample. Neither did the prediction accuracy improve after selecting only males or females. In both cases, prediction accuracy was 60%.

## 4 Discussion

### 4.1 Overview of main results

With SVM, a trained anatomical classifier correctly classified 80% of students with and without dyslexia (82% of students with dyslexia; 78% of students without dyslexia; d-prime = 1.67). Regions that were important in discriminating between these groups were the LOFG and the ROFG and the LIPL. Severity of dyslexia was defined with mean classification accuracy and correlated positively with severity of dyslexia according to behavioural measures (r = 0.47). We found six significant correlations between the three regions and behavioural measures of dyslexia. In an independent sample of a general population, the anatomical trainer of the first sample correctly classified 59% of young adults with and without dyslexia (67% of students with dyslexia; 59% of students without dyslexia; d-prime = 0.65).

### 4.2 Evaluation of prediction accuracy

In a well-balanced sample of students with and without dyslexia, a majority was correctly classified using SVM. In a general population sample, the trained classifier of the first sample resulted in a much lower classification performance, but still above chance. The advantage of using this classification approach over traditional analyses of group differences is that discussions about statistical corrections for multiple comparisons are not relevant. The statistical significance of prediction accuracy of the anatomical classifier was supported by a cross-validation approach and by a low *p*-value (< 0.001). The reliability of the classifier was supported by the finding that on average, subjects were classified with high consistency between trials, which was expressed by a mean classification accuracy of 0.89. The validity of the classifier was supported by the finding that this mean classification accuracy correlated positively with a measure of behavioural severity of dyslexia. The reliability of the anatomical classifier was further confirmed in a second sample, although prediction accuracy and calculated d-prime were much lower in the second sample than in the first sample. This decline resulted mainly from many false alarms.

From a diagnostic point of view, predictive values are useful measures. These measures are, however, sample specific. In the first sample, positive and negative predictive value are high but meaningless because the equal groups of the first sample do not represent reality with a prevalence of dyslexia of 5–15%. However, we still can draw meaningful conclusions. We can imagine a sample with a more representative fraction of students with dyslexia, for instance, with the number of students without dyslexia being ten times higher than the 27 students in the first sample. Classification performance would then approach specificity, which is 78%, only 2% below the overall classification performance of 80%. However, positive and negative predictive value would change. Negative predictive value would then be 98%, meaning that a prediction of no dyslexia by the classifier is in most cases correct. In contrast, positive predictive value would be 23%, meaning that in about one of four cases a prediction of dyslexia is correct, but incorrect in three out of four cases. How to explain this large number of false alarms? Analyses of mean classification accuracy revealed that not only the correctly classified students but also false positives and false negatives were consistently classified by the trainer in most of the cases (90%). Apparently, the anatomical features represented by the classifier may represent something else than dyslexia in some of the cases.

In the second sample, which represented a general population, we found a much lower classification performance of 59%. Although negative predictive value was high (96%), meaning that most people who are classified as not dyslexic are indeed not dyslexic, positive predictive value was very low (11%), also lower than in the first sample. This resulted from a high percentage false alarms of 43%, much higher than estimations of prevalence of 5–15%. Before drawing conclusions about the generalizability of the trained classifier, we should discuss three issues.

First, the first sample was small and consisted of equal groups with subjects only being selected when having dyslexia or no dyslexia beyond any reasonable doubt, while the second sample was large and represented a general population. Generally, it is widely accepted that subtypes of dyslexia can be distinguished (e.g. Ramus and Ahissar, 2012). Although we found that the students with dyslexia in the first sample can be characterized by five different impairments, we cannot exclude the possibility that in such a small sample of a specific subpopulation (first-year college students) one or more cognitive aspects of dyslexia are over- or underrepresented as compared to a general population. If either of these possibilities would have been the case, the trained classifier was trained on a subgroup of people with dyslexia. This might have compromised classifications in both samples. For instance, specific subtypes of dyslexia may be characterized by specific compensation strategies with specific anatomical consequences. It cannot be ruled out that also students without dyslexia are characterized by the same anatomical consequences because their training histories during school days resemble those of students with dyslexia.

A second issue is the criterion of dyslexia in the second sample. This criterion was established based on diagnoses during school days by specialists. But these records were not specified and were uncontrollable. Based on this criterion, we found a prevalence of dyslexia of seven percent, which may be too low. It can be assumed that some students without records of dyslexia still have dyslexia, while some students with an official certificate of dyslexia have no dyslexia. Although it cannot explain the large number of false alarms, overall classification performance could have been better with better criterion groups.

A third issue is that the two samples were different regarding intelligence, socio-economical status, or other characteristics. One clear difference between students of a university and other young people is that students have received more training than other young people in all kinds of language-related and other cognitive abilities. And in the Netherlands, school children with dyslexia usually receive additional remedial teaching. When we hypothesize that the anatomical differences of the anatomical trainer in this study partly result from training effects, it might be explained why the number of false alarms in the second sample was larger than in the first sample. Subjects without dyslexia, but with low socio-economical status or low intelligence, may have received additional training as well, resulting in a diagnosis of dyslexia in this study. The hypothesis that the anatomical trainer in this study results from training differences is supported by various studies showing effects of training on anatomical alterations in dyslexia (e.g. Hoeft et al., 2011, Krafnick et al., 2011). Two studies report that many GM volume differences between dyslexics and controls in general result from differences in reading experience (Clark et al., 2014, Krafnick et al., 2014).

In short, the classifier found in the first sample performed above chance in the second sample. This underlines its reliability and justifies its further theoretical examination of the areas that contributed to this classifier. However, we conclude that the trained classifier based on anatomical scans of students of this study cannot be used for clinical purposes. Although negative predictive values were high in both samples, positive predictive values were low in both samples. This means that many people without dyslexia would be labelled with dyslexia.

### 4.3 Anatomical classifier

While the usefulness of a trained classifier based on anatomical scans for clinical purposes requires further examination in future studies, the nature of the classifier in this study provides useful information as compared to previous results of brain imaging studies. In the present study, brain regions that contributed to the classification were found in the LIPL, the LOFG and ROFG. These results are in line with converging evidence of involvement of these areas in dyslexia. For instance, in the classification study of Tanaka et al. (2011), poor reading children exhibited significantly reduced activations in the LIPL and the LOFG during phonological processing. We will discuss the areas of the present study one by one.

In the present study, GM volume in the LIPL correlated positively with performances of spelling and phonology, which is consistent with various previous findings. Reduced GM volume in the LIPL has been reported in pre-reading children with a family-history of developmental dyslexia (Raschle et al., 2011). These researchers suggested that some structural alterations in developmental dyslexia may be present at birth or develop in early childhood prior to reading onset. They also found a significant positive correlation between this area and a rapid automized naming test, which is assumed to be related to phonological skills (Vaessen et al., 2009, Vaessen and Blomert, 2010) and which is reported to be one of the main precursors of later reading ability in children (e.g. De Jong and Van der Leij, 1999). These results suggest that some anatomical differences related to phonology in the LIPL may be present already at birth. Furthermore, the LIPL has been reported in functional brain imaging studies that showed that multiple specializations along the visual word-form system were found to be impaired in dyslexics (Van der Mark et al., 2011), which is consistent with the reduced activations found in the study of Tanaka et al. (2011).

While in the present study dyslexics exhibited less GM volume in the LIPL, they exhibited more GM volume in the LOFG and ROFG. And although GM volume in both areas correlated (negatively) with severity of dyslexia, only the LOFG correlated with behavioural measures: negatively with whole-word reading and negatively with spelling (in contrast to the positive correlation found between the area in the LIPL and spelling). These negative correlations are remarkable: better performances on whole-word reading and spelling are accompanied by reduced GM volume. Maybe this should be interpreted as the result of training effects with poor performances leading to more training and thus to augmented GM volume. Interesting here is also the finding that poor reading children exhibited reduced activations in the LOFG during phonological processing (Tanaka et al., 2011), while no significant correlation was found between this area and phonology in the present study.

Clearly, the relation between GM volume and functionality in the LOFG is hard to understand. However, it is also clear that the LOFG is an important area in dyslexia. In previous studies, support was found for the involvement of the LOFG in dyslexia, but mainly in the VWFA. In the present study, the area in the LOFG is located close to where the VWFA is usually reported, although we were not able to establish whether this area is actually the VWFA. Nevertheless, the correlations between the LOFG and spelling and whole-word reading are consistent with previous findings related to the VWFA. For instance, various studies reveal that the VWFA plays an important role in early stages of whole-word recognition and serial sublexical coding of letter strings (Dehaene and Cohen, 2011, Glezer et al., 2010, Schurz et al., 2010). Furthermore, lesions in the VWFA cause pure alexia, a selective deficit in word recognition characterized by a disproportionate prolongation of reading time as a function of word length (Pflugshaupt et al., 2009). Possible training effects are supported by showing that the category-selective nature of the VWFA for visually presented words is dependent of experience with specific orthographies (Baker et al., 2007).

### 4.4 Anatomy and functionality

Although the brain areas found in this study can be related to previous findings, interpretations are hard to make. In general, dyslexics are found to have less GM volume in various areas, but some studies report more GM volume in some areas (Silani et al., 2005, Vinckenbosch et al., 2005). Likewise, reduced as well as augmented activations have been reported in the literature (e.g. Richlan et al., 2011). Some cognitive aspects of dyslexia that are typically impaired in people with dyslexia (e.g. phonological awareness, visual/attentional processing) correlate and others do not correlate with brain volume or activation.

In this study, we found a relationship between three behavioural measures of dyslexia (spelling, phonology and whole-word reading) and brain anatomy but no correlations for short-term memory and visual/attentional confusion, confirming results of previous studies showing that different symptoms of dyslexia exist at different levels of brain organisation. A complicating factor is that anatomical and functional differences may change in the course of a lifetime as the result of differences in training. Another complicating factor is that subgroups of dyslexia exist that suffer from different types of symptoms (e.g. Bosse et al., 2007), which may be related to differences between languages, differences in socioeconomic status, differences in intelligence or differences in schooling.

For example, previous studies have shown that some aspects of dyslexia appear to be genetically induced (e.g. Carrion-Castillo et al., 2013), while other aspects are related to the development of cognitive abilities and training effects in general (e.g. Clark et al., 2014, Hoeft et al., 2011, Krafnick et al., 2011, Krafnick et al., 2014). This might result in puzzling findings such as those in the classification study of Pernet et al. (2009). It was found that voxels in the right cerebellar declive and in the right lentiform nucleus classified subjects with and without dyslexia correctly. Remarkably, regarding the cerebellar declive two subtypes of dyslexics could be distinguished: one subtype having more GM volume than controls, and one subtype having less GM volume than controls. After behavioural analyses, the researchers found that these brain phenotypes relate to different deficits of automatization of language-based processes. Thus, it even may be the case that different subgroups of people with dyslexia are characterized by different training histories, either induced by additional training programs or by compensation strategies.

Even more complicating for the interpretation of the relation between brain measures and behavioural measures of dyslexia is that we found both positive and negative correlations between behavioural measures and the brain. For instance, good spelling performances correlated positively with GM volume in the LIPL, but negatively with GM volume in the LOFG. In general, it is hard to interpret the difference between positive and negative correlations because the relation between brain anatomy and functionality remains unclear. The negative correlations in our study may point to effects of more training of people with dyslexia in comparison with people without dyslexia. Especially in our student sample, it might be expected that the students with dyslexia were encouraged to participate in remedial teaching programs during childhood, because dyslexia tends to be more disturbing when the discrepancy with intelligence is high. Support for this view can be found in a recent study in which fMRI was used to investigate the extent of anatomical overlap between three neural systems which are associated with dyslexia in the literature: the auditory phonological, the visual magnocellular and the motor/cerebellar systems (Danelli et al., 2013). Various areas of conjunction were found in the occipito-temporal cortex at more or less the same locations as our two areas in the LOFG and ROFG.

In short, we found that both augmented and reduced GM volumes contribute to an anatomical classifier. Possibly, reduced GM volume in the LIPL may be caused by genetic influences while augmented GM volume is related to training effects which differ between students with and without dyslexia, but also between various subtypes of students with dyslexia and between students without dyslexia who are characterized by different training histories. We also found both positive and negative correlations between these areas and behavioural measures. The sample that was used for creating the classifier consisted of well-educated students which might have influenced the classifier with regard to training effects. It is unknown to what extent all subgroups of dyslexia were represented by the classifier in a balanced way. Nevertheless, the areas that contributed to the classifier are consistent with various findings from previous studies. Observations made in this and other studies not only show that different aspects of dyslexia exist at different levels of neural organisation but also that dyslexia is not a unified phenomenon. Dyslexia results from an interplay between anatomy and functionality which result from both genetics as training effects, while it also should be accounted for that different subtypes of dyslexics exhibit different combinations of anatomy and functionality.

### 4.5 Conclusion

In summary, we report prediction accuracy of dyslexia using machine learning of anatomical scans in two samples. Various predictive values showed that the anatomical classifier of this study is still far away from use in clinical settings. However, the areas that contributed to the classification of students with and without dyslexia contribute to the understanding of brain anatomy in dyslexia. We concluded that relations between brain anatomy and functionality are hard to interpret, especially when considering effects of training and the existence of subtypes of dyslexia. We furthermore concluded that not all symptoms of dyslexia exist at the same cortical level of organisation, suggesting not only that dyslexia is not a uniform syndrome, but also illustrating that these multi-variate techniques can be used to arrange and evaluate the symptoms that are believed to belong to a syndrome. Findings in this and previous studies may give directions to further research for suitable biomarkers in the future that can be used in a clinical setting.

## Figures and Tables

**Table 1:** Classification of subjects with and without dyslexia.

Sample 1 (N = 49)	Predicted as	
Dyslexic	Not dyslexic
Subjects with dyslexia	18	4
Subjects without dyslexia	6	21
Prediction accuracy	80% (*p* < 0.001)	
d-Prime	1.67	
Sensitivity	82%	
Specificity	78%	
Positive predictive value	75%	
Negative predictive value	84%	


Sample 2 (N = 876)	Predicted as	
Dyslexic (43%)	Not dyslexic (57%)
Subjects with dyslexia (7%)	40	20
Subjects without dyslexia (93%)	338	478
Prediction accuracy	59% (*p* = 0.07)	
d-Prime	0.65	
Sensitivity	67%	
Specificity	59%	
Positive predictive value	11%	
Negative predictive value	96%	

**Table 2:** GM regions discriminating between subjects with and without dyslexia.

Anatomical region	MNI-coordinates (x, y, z)			Cluster size (mm^3^)	N voxels	Direction of effect
Left occipital fusiform gyrus (visual word form area)	− 35	− 72	− 21	1200	150	Dyslexic > control (*p* = 0.02)
Right occipital fusiform gyrus	35	− 67	− 19	1496	187	Dyslexic > control (*p* = 0.01)
Left inferior parietal lobule	− 53	− 28	24	520	65	Control > dyslexic (*p* = 0.21)

**Table 3:** Sample 1: Pearson correlations (whole group: N = 49) of brain indices with measures and severity of dyslexia (severity of dyslexia — low score).

	Right occipital fusiform gyrus	Left occipital fusiform gyrus	Left inferior parietal lobule
Spelling	− 0.267	**− 0.311** **(*p*** **=** **0.029)**	**0.322** **(*p*** **=** **0.024)**
Phonology	− 0.242	− 0.134	**0.301** **(*p*** **=** **0.036)**
Short-term memory	− 0.177	− 0.215	0.222
Visual/attentional confusion	− 0.152	− 0.137	0.128
Whole-word reading	− 0.239	**− 0.318** **(*p*** **=** **0.026)**	0.208
Severity of dyslexia	**− 0.377** **(*p*** **=** **0.008)**	**− 0.337** **(*p*** **=** **0.018)**	0.224

Bold values indicate significance at p < 0.05.

## Appendix A Supplementary data

Supplementary material. Image 1
