# Supplementary material for: Machine learning and dyslexia: Classification of individual structural neuro-imaging scans of students with and without dyslexia
Source: Neuroimage Clin. 2016 Mar 29;11:508–14. doi: 10.1016/j.nicl.2016.03.014 (PMC4832088; doi:10.1016/j.nicl.2016.03.014)

**Legends for figures:**

**Figure 1:**

Classification Scheme

**Figure 2:**

Regions with voxels involved in discriminating between subjects with and without dyslexia. Post hoc analysis revealed that the region in blue (LIPL) is smaller in subjects with dyslexia and that the regions in red (LOFG and ROFG) are larger in subjects with dyslexia.

**Supplementary Information Sample 1**

**Group differences on school grades, intelligence and five elements of dyslexia**

___________________________________________________________________________

Dyslexia No dyslexia

(N=22) (N=27)

Mean SD Mean SD T *p*

___________________________________________________________________________

**Final School Grades**

Dutch 6.50 0.89 7.16 0.75 2.71 0.010

English 6.71 1.01 6.92 1.32 0.59 0.562

Other Languages 6.61 0.70 7.15 0.58 2.77 0.008

Mathematics 6.19 1.66 6.41 0.73 0.55 0.587

Other Courses 6.95 0.59 7.00 0.55 0.27 0.788

__________________________________________________________________________

**Intelligence (factor scores, normalized to a student population)**

Non-verbal -0.28 0.86 -0.06 0.98 0.74 0.466

intelligence

Speed of numeric -0.65 0.62 -0.30 0.82 1.52 0.136

processing

Vocabulary -0.54 1.02 -0.03 1.00 1.62 0.113

___________________________________________________________________________

**Elements of Dyslexia (Z-scores)**

Spelling -1.60 0.65 0.54 0.79 10.23 < 0.001

Phonology -1.23 1.02 0.33 0.86 5.77 < 0.001

Short-term -1.32 0.78 0.42 0.99 6.71 < 0.001

memory

Visual/attentional -0.62 0.66 0.49 0.82 5.12 < 0.001

Confusion

Whole-word -1.23 0.71 0.43 0.78 7.70 < 0.001

reading

___________________________________________________________________________

**Scatter plots of correlations between GM volume and behavioural measures**


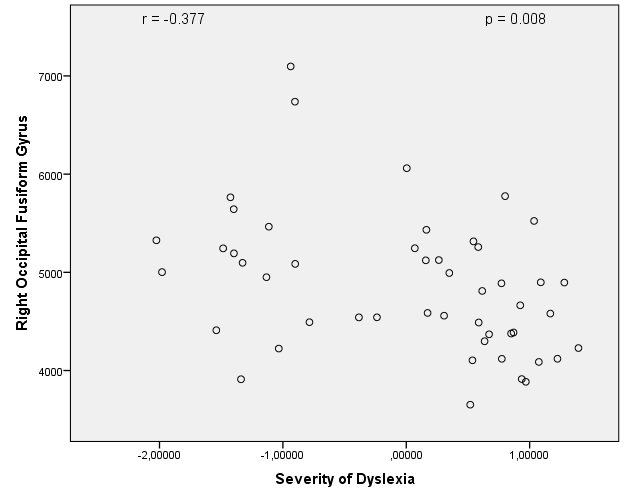


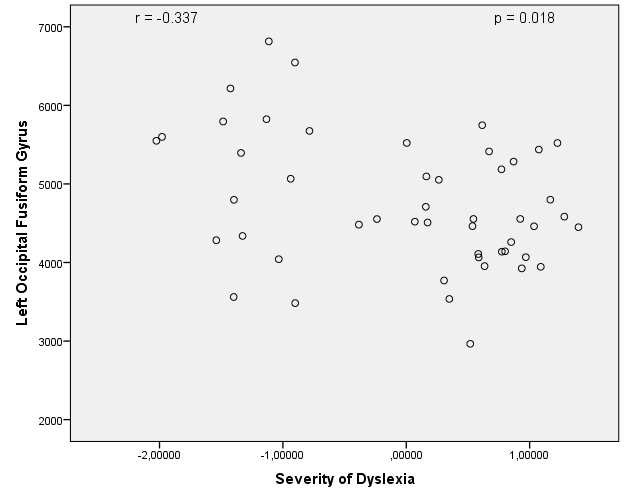


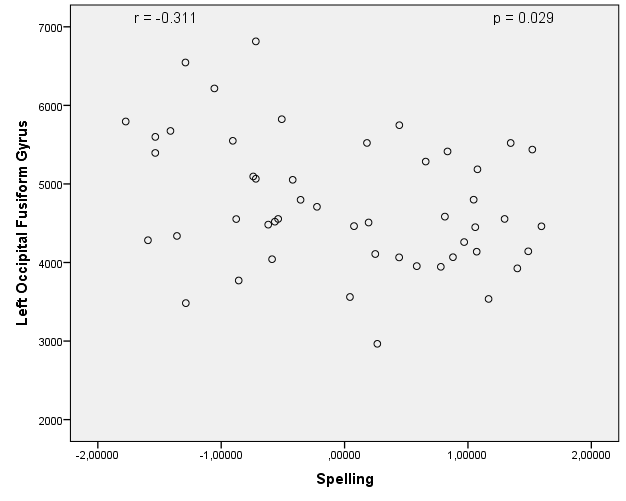


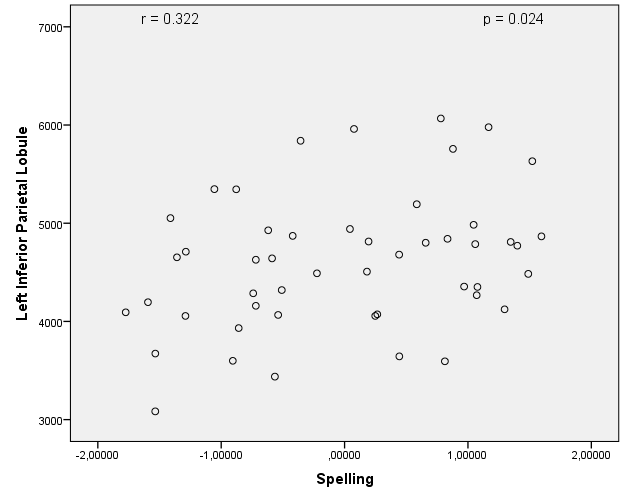


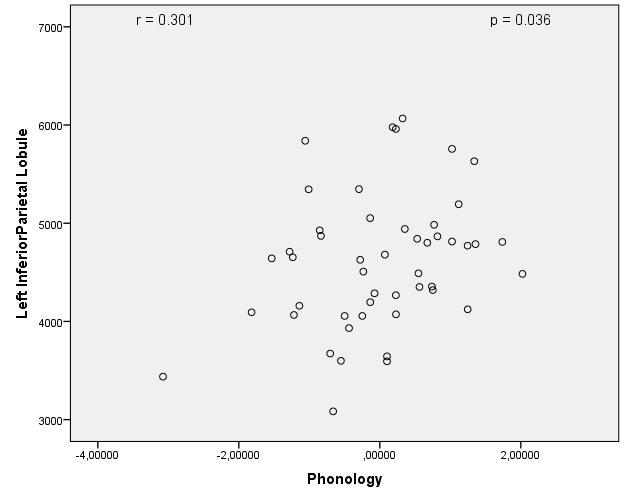


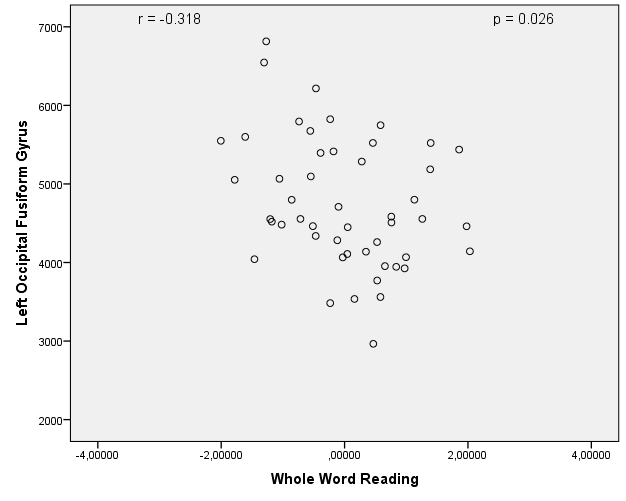

Supplement: Supplementary file 1 — Supplementary material. [file mmc1.doc]
